# Binasal congruous hemianopia secondary to functional visual loss: A case report

**DOI:** 10.1097/MD.0000000000020754

**Published:** 2020-07-02

**Authors:** Georgios Tsokolas, Hina Khan, Straton Tyradellis, Jithin George, Mark Lawden

**Affiliations:** a Ophthalmology Department; b Department of Neurology, Leicester Royal Infirmary, Leicester, LE1 5WW, United Kingdom.

**Keywords:** electroretinogram, functional visual loss, Goldmann visual fields, Humphrey visual fields, optical coherence tomography, retinal nerve fiber layer, visual evoked potentials

## Abstract

**Introduction:**

To describe an unusual case of binasal congruous hemianopia secondary to functional visual loss (FVL).

**Patient concerns:**

A 24 year-old male was referred originally by his optician at the Emergency Eye Department of the Leicester Royal Infirmary in October 2018 with visual field changes affecting the nasal field of vision in both eyes on routine eye examination. The patient reported ongoing headaches over the last 6 weeks to 8 weeks associated with simultaneous peripheral visual field changes. He also reported rapid loss of weight over the same period of time.

**Diagnosis:**

Binasal congruous hemianopia secondary to FVL.

**Interventions:**

Full past medical and ocular history was obtained. The patient underwent full ophthalmic examination including dilated fundoscopy. Visual acuity was recorded with Snellen Chart. Color vision was assessed with Ishihara plates. Peripheral vision was assessed with both Humphrey visual fields and Goldmann visual fields. Optical coherence tomography of the macula and discs was also performed. Neuroimaging investigations included Computerized Tomography (CT) and Magnetic Resonance Imaging (MRI) of the Brain. Electrophysiology investigations included Electroretinogram and visual evoked potentials. Patient was also tested for syphilis.

**Outcomes:**

Humphrey visual fields and Goldmann visual fields confirmed the presence of complete binasal field defects. Optical coherence tomography, electroretinogram, visual evoked potentials, CT, MRI were all unremarkable. Ocular examination was normal. Finally, syphilis serology was negative. After 1 year of follow-up, the visual field changes have remained the same.

**Conclusion:**

To the best of our knowledge, this is the fourth case described in the literature with complete congruous binasal hemianopia due to FVL. We advocate thorough investigations with multimodal imaging of the fundus, neuroimaging and syphilis serology to exclude serious organic causes for binasal field defects prior to labeling such a field defect functional. Such patients may benefit from neuropsychological input to understand the psychological factors that may be contributing to the symptoms.

## 1 Introduction

We are writing this brief report to describe a case of binasal hemianopia secondary to functional visual loss (FVL). Binasal hemianopia is an unusual visual field defect that is rarely encountered by both neuro-ophthalmologists and neurologists. There have been a few cases in literature with binasal hemianopia with an identifiable organic cause^[1–6]^ and 3 cases with no obvious identifiable organic cause.^[7,8]^ In the first 5 referenced manuscripts, the binasal field defects were incomplete,^[1–5]^ whereas in the sixth the binasal defects were complete.^[6]^ In the 3 cases with no obvious identifiable organic cause, the binasal field defects were complete as well.^[7,8]^ In this manuscript, we describe another case of complete congruous binasal hemianopia with an otherwise normal ocular examination, normal neuroimaging and normal multimodal imaging of both retinas and optic discs. To the best of our knowledge, this is the fourth case of complete binasal hemianopia without any obvious underlying organic cause described in literature.

## 2 Case presentation

A 24 year-old male was referred originally by his optician at the Emergency Eye Department of the Leicester Royal Infirmary in October 2018 with visual field changes affecting the nasal field of vision in both eyes on routine eye examination. The patient reported ongoing headaches over the last 6 weeks to 8 weeks associated with simultaneous peripheral visual field changes. He also reported rapid loss of weight over the same period of time. He denied any other significant neurological deficit. He had no past medical or ocular history of note. The only pertinent information from his social history was that he used to have more than 1 sexual partner. He attended the Genitourinary Medicine Clinic, where he underwent full screening for sexually transmitted diseases, which was negative. He used to take paracetamol and codeine on an as required basis for the headaches and he had no known drug allergies.

**Figure 1:**
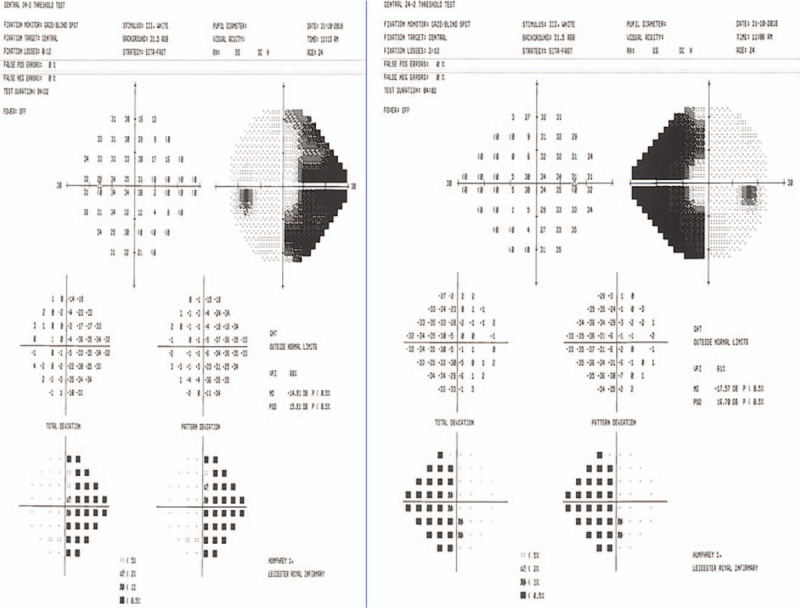
Humphrey visual fields 24–2 on presentation in Eye Casualty.

**Figure 2:**
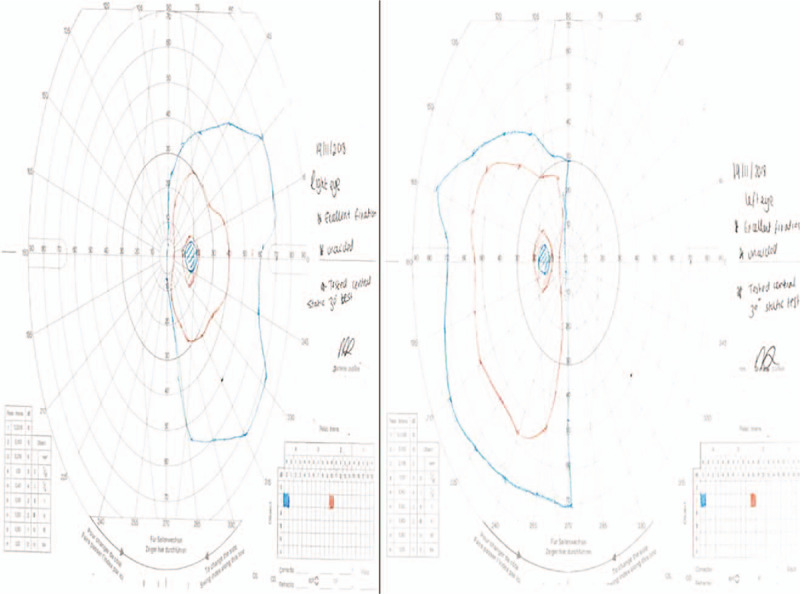
Goldmann visual fields on initial presentation.

**Figure 3:**
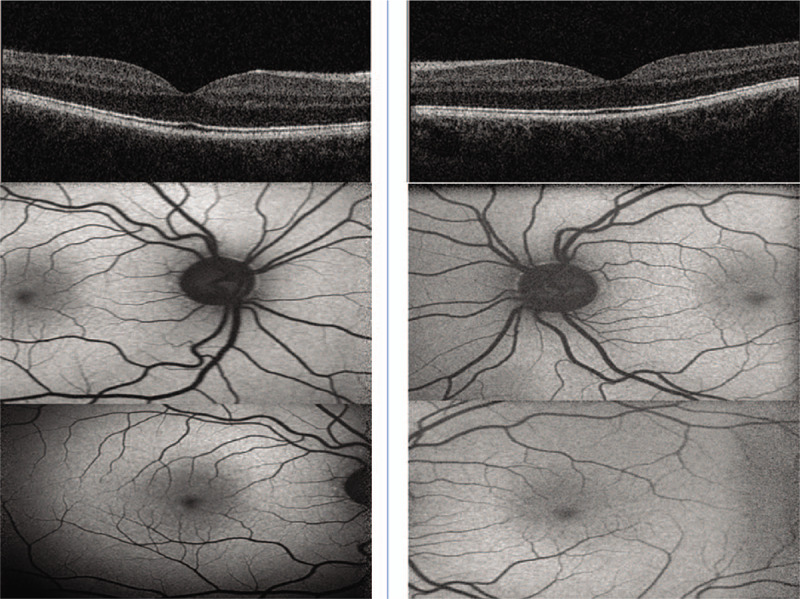
OCT scans and Fundus Autofluorescence of both eyes. Left side represents the right eye and the right side the left eye. A) Top Row: OCT maculae of both eyes B) Middle Row: Disc Autofluorescence C) Bottom Row: Macula. OCT = optical coherence tomography.

**Figure 4:**
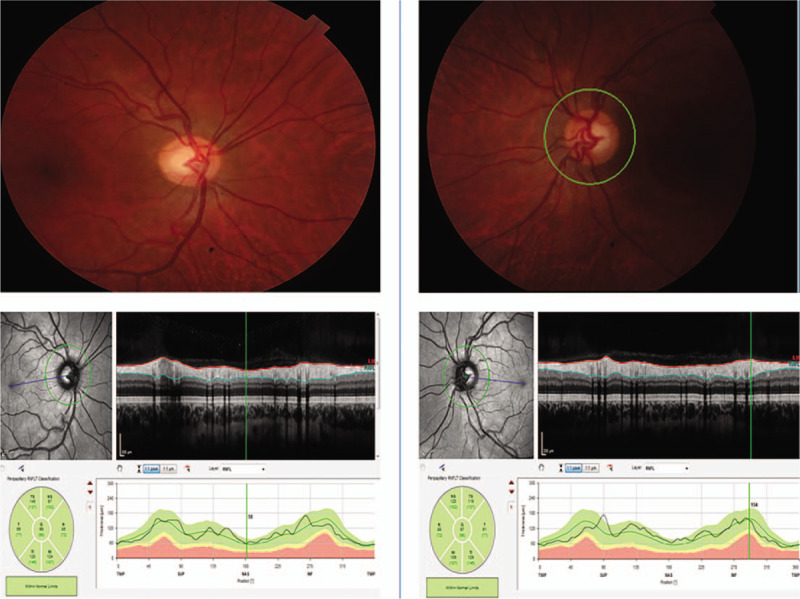
Color photographs and retinal nerve fiber layer (RNFL) of both discs. Left side represents the right eye and the right side the left eye. As shown, both color photographs show healthy looking discs, normal posterior pole and normal RNFL. RNFL = retinal nerve fiber layer.

On examination, his best corrected visual acuity was 6/5 with the Snellen chart in both eyes. Both pupils were equal and responding to light without any afferent pupillary defect. Color vision was normal. Humphrey visual fields (Humphrey Field Analyzer, Carl Zeiss Meditec, Dublin, CA) and Goldmann Visual Fields (GVFs) did confirm the presence of complete binasal field defects (Figs. 1 and 2). Anterior segment examination was within normal limits. Dilated fundus examination revealed bilaterally normal discs, maculae and peripheral retinas. Multimodal imaging of both eyes including wide field fundus autofluorescence, optical coherence tomography of both macula and retinal nerve fiber layer was completely normal (Figs. 3 and 4). Images were obtained with the Heidelberg Eye Explorer (Heidelberg Engineering Germany, Version 1.9.10.0, 2014).

**Figure 5:**
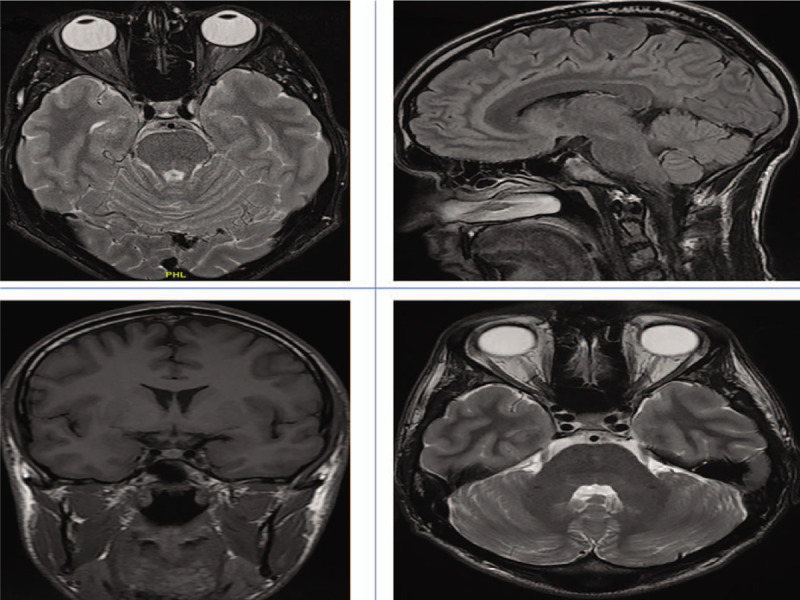
Cumulative Image of magnetic resonance imaging scan: Top row: A) Transverse section B) Sagittal section. Bottom row: C) Coronal section D) Transverse section. All sections revealed no structural abnormalities.

Given the history and clinical findings and with no obvious intraocular cause for the field defects being identified, the patient was referred urgently to the Neurology Team for admission. He underwent originally a computerized tomography with intravenous contrast of his brain which was normal. He subsequently underwent an urgent magnetic resonance imaging combined with angiography, which was also normal (Fig. 5A, B, C, D). In addition, syphilis serology was also repeated and was negative. The rest of the blood tests did not reveal any signs of inflammation. The results of all these investigations were reported to the patient. He was offered lumbar puncture. However, the patient decided against this and self-discharged.

**Figure 6:**
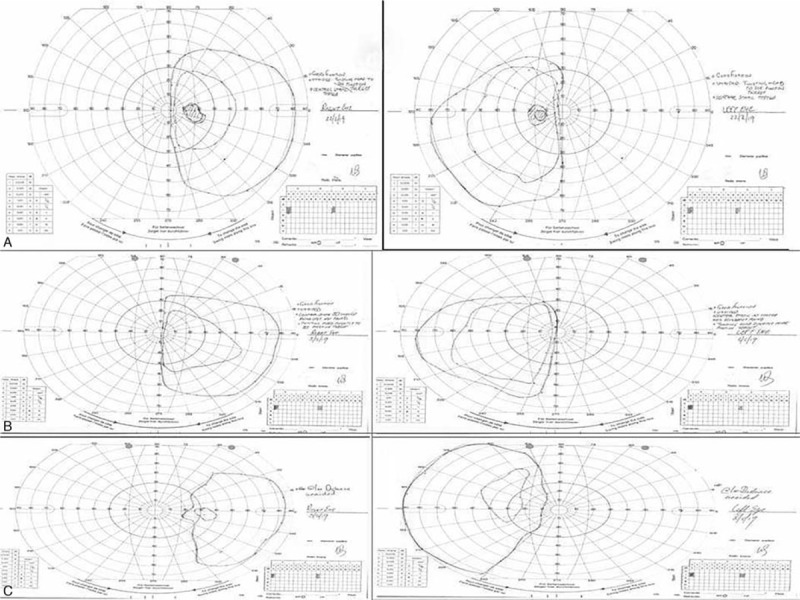
Cumulative image of the repeated Goldmann visual fields (GVFs). A) Top row: Goldmann visual fields (GVFs) performed in February 2019 B) Middle Row, C) Bottom Row: Goldmann Visual Fields (GVFs) performed in May 2019 at 2 different distances to assess for functional visual loss. Mapping of the peripheral visual fields in both eyes revealed persistent binasal hemianopia. However, note that there are differences in the patterns of the binasal defects which suggest a functional peripheral visual field loss.

**Figure 7:**
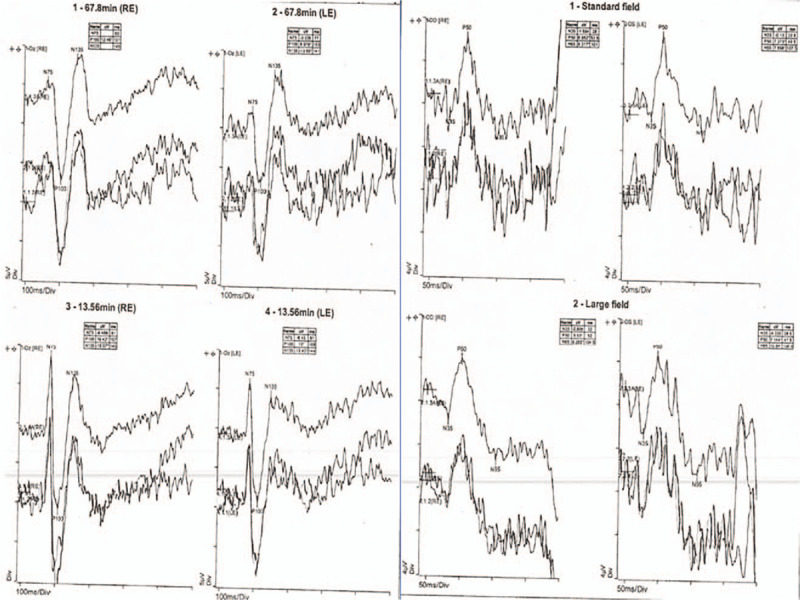
Electrophysiological investigations: Left side: A) Visual evoked potentials (VEPs). Right side: B) Electroretinogram (ERG). Both tests revealed normal responses and could not explain the persistent binasal field defects. VEP = visual evoked potentials.

A follow-up in the Neuro-ophthalmology clinic was arranged approximately a month after the patient decided to self-discharge. His visual acuities were 6/9 on the Snellen chart. The pupil reflexes were still normal and the color vision was still intact. Dilated fundoscopy revealed healthy optic discs and maculae bilaterally. The patient, however, was still complaining about binasal field defects. The GVFs were repeated again twice. The second time they were performed at 2 different distances as FVL was suspected given the previous normal neuroimaging (Fig. 6 A, B, and C). The GVFs revealed bilateral persistent binasal hemianopia. However, the patterns of the field defects in the different GVFs attempts were not quite similar and this enhanced the suspicion of functional peripheral visual field loss. Furthermore, the electrodiagnostic tests performed including visual evoked potentials and electroretinogram were also normal (Fig. 7 A, B).

During his follow-up visit in May 2019, the patient was still complaining about binasal field defects. His visual acuities on the Snellen chart were 6/12 and 6/9 right and left eye respectively. He also had reduced color vision on the Ishihara plates in both eyes reading 10 out 17 numbers with the right eye and 11 out of 17 plates with the left eye. The anterior segment examination revealed bilateral dry ocular surface for which the patient was prescribed topical lubricants. However, this cannot account for his binasal field defects. Repeated dilated fundoscopy revealed bilaterally healthy optic discs with pink and healthy neuroretinal rims and no signs of swelling or pallor.

A follow-up was arranged in the beginning of December 2019 in the Combined Neurology and Neuro-ophthalmology clinic that takes place in Leicester Royal Infirmary once every month but the patient did not attend his scheduled appointment for whatever reason. We are planning to review the patient again with a view of considering further referral to neuropsychology.

## 3 Discussion

Binasal hemianopia is a rare and very unusual field defect. Organic causes of this unusual field defect include brain space occupying lesions, keratoconus, bilateral internal carotid aneurysms, neurosyphilis, optic neuropathy, optic disc drusen and optic nerve pits, retinitis pigmentosa sine pigmento, as well as pneumosinus dilatans of the sphenoid sinuses.^[1–6]^ In binasal hemianopia with an identifiable organic cause, the field defects were incomplete except in the case of pneumosinus dilatans of the sphenoid sinuses, where the defects were complete. In our case, the field defects were also complete as shown in Figure 1. In addition, our patient had normal neuroimaging and multimodal imaging of both fundi. Thus, in our case, no intraocular or intracranial causes for the binasal hemianopia were identified.

After an extensive literature review, there are only 2 other cases described that developed complete binasal hemianopia without any obvious intraocular or intracranial cause identified.^[7]^ Both patients were female, whereas our patient was male. Similar to the 2 reported cases, we could not identify any intraretinal cause for such congruous binasal field defects that respected the vertical midline. Dilated fundal examination did not reveal any lesions in the peripheral retina that could account for the visual field defect. This was also supported by the wide field autofluorescence. Furthermore, both discs looked normal on dilated fundus examination without any signs of diffuse or sectorial pallor. The disc optical coherence tomography supports our clinical findings. Our patient had initially better than 6/6 initial visual acuity, normal color vision and no lens opacities. However, in his last follow-up his visual acuities were reduced to 6/12 and 6/9 right and left eye respectively, with decline in color vision. Nevertheless, his pupillary reflexes were normal and dilated fundoscopy of both eyes revealed healthy discs, maculae and normal peripheral retinas. The electrodiagnostic tests and neuroimaging studies were normal, therefore we cannot identify any obvious underlying organic cause for the patient's symptoms.

In addition, there is 1 more case described in literature of a 48 year-old male patient who developed binasal hemianopia which evolved into homonymous hemianopia after a head injury and subsequent concussion.^[8]^ His neuroimaging was normal and the visual field defect was attributed to FVL. In our case, however, there was no history of head trauma and the field defect did not transform to homonymous hemianopia but remained as it was on initial presentation.

Bedside visual field examination by confrontation was also performed by the Neurology Team, but the findings did not match the Humphrey visual fields and GVFs. Careful examination of his visual fields by confrontation in each eye revealed a binasal hemianopia respecting the midline precisely yet when he simply looked at the examining doctor's face binocularly he claimed not to be able to see it. As his eyes were straight in the primary position the field midlines in each eye should have corresponded so there should have been no central binocular scotoma at all, let alone the large 1 that he insisted he experienced. Therefore, we propose that our patient developed a functional visual field loss and was potentially malingering. Further investigation with a lumbar puncture would have been useful to exclude inflammatory conditions, but as mentioned the patient declined.

In the cases reported in the literature, a congenital cause for the binasal hemianopia was postulated, with proposal of a defective organization of the temporal retina fibers resulting in manifestation of these congruous binasal defects, therefore affecting the visual pathway posteriorly to the lateral geniculate nucleus.^[6]^ However, we do not advocate the same hypothesis for our case. Our diagnosis secondary to all normal investigations was likely functional with no organic pathology being identified. Such cases can benefit from neuropsychological input to understand the psychological factors that may be contributing to the symptoms.

After reviewing the literature and to the best of our knowledge, this the fourth case described in literature of complete congruous binasal hemianopia secondary to functional non-organic cause. Our point of learning is that such diagnoses are diagnoses of exclusion. We therefore advocate thorough investigations with multimodal imaging of the fundus, neuroimaging and syphilis serology to exclude serious organic causes for binasal filed defects prior to labeling such a field defect functional.

## Author contributions

Dr. Khan: Participated in the drafting of the original manuscript.

Dr. Tyradellis, Dr. George and Dr. Lawden: Editing and preparing the figures and their legends, correction of the original draft of the manuscript and also literature review.
